# Closed suction drains, reinfusion drains or no drains in primary total knee replacement?

**DOI:** 10.1308/003588412X13171221590098

**Published:** 2012-07

**Authors:** S Al-Zahid, AP Davies

**Affiliations:** Abertawe Bro Morgannwg University Health Board,UK

**Keywords:** Total knee arthroplasty, Autologous transfusion, Reinfusion drains, Haemoglobin, Blood transfusion

## Abstract

**INTRODUCTION:**

Controversy still surrounds the use of drains after total knee replacement (TKR). We compared closed suction drains, reinfusion drains and no drains by studying haemoglobin (Hb) levels, blood transfusion requirements and functional knee outcome scores in a single surgeon series.

**METHODS:**

A total of 102 consecutive primary TKRs were performed by the senior author between September 2006 and July 2008. All were cemented fixed bearing devices with patellar resurfacing. Of the 102 patients, 30 had closed suction drainage, 33 had an unwashed reinfusion drainage system and 39 had no drains. Data regarding pre and post-operative Hb and units transfused were gathered retrospectively. Pre and post-operative American Knee Society scores (AKSS) and Oxford knee scores (OKS) were recorded prospectively.

**RESULTS:**

The pre-operative Hb levels were comparable among the groups. There was no statistically significant difference in Hb level reduction or autologous transfusion rates among the groups. Pre-operative AKSS and OKS were statistically comparable in each group. There was no statistical difference between the improvement in AKSS knee and function scores in all three groups. There was a slightly smaller improvement in the OKS of the ‘no drain’ group. There were no complications of drain usage and no deep infections. No patient required manipulation under anaesthesia and range of movement outcomes were the same for each group.

**CONCLUSIONS:**

Our study does not support the use of either closed suction drains or reinfusion drains in primary elective TKR.

Primary total knee replacement (TKR) can result in a considerable amount of blood loss.^1^ A number of strategies to reduce the need for allogenic red cell transfusion have been employed such as the use of thigh tourniquets,^2^ diathermy coagulation, knee positioning,^3^ clamping drains,^4^ adrenaline and saline infiltration,^4^ and computer assisted navigated TKRs.^1^ Most bleeding in TKRs occurs post-operatively. Drains in arthroplasty have been used historically for the theoretical benefit of preventing wound haematoma, improving wound healing and preventing infection. More recently, retransfusion drains have been used to reduce the need for allogenic blood transfusion.^5^ This is fuelled by the many risks involved: transmission of blood borne infections (such as HIV, hepatitis B and C, and Creutzfeldt–Jakob disease), transfusion related reactions, blood group transfusion errors and the high cost of allogenic transfusion.^5^

The purpose of our study was to address the question of what is the best post-operative drainage regime after primary TKR: a closed suction drain, a retransfusion drainage system or no drain at all. A review of the literature found only one previous study that had compared each of these three treatment groups directly.^6^ Our aim was to repeat that study in a single surgeon series to minimise confounding variables. Outcome was measured in terms of blood loss, transfusion requirements and outcome scores from the procedure.

## Methods

A total of 102 consecutive unilateral primary TKR procedures were performed between September 2006 and July 2008 by the senior author. Revision knee arthroplasties and unicompartmental knee arthroplasties were excluded. All procedures were performed or directly supervised by the senior author using a standardised technique.

All procedures used a high thigh tourniquet, a midline skin incision and a medial parapatellar approach. All patellas were resurfaced and all components cemented. Three prostheses were used during the study: Scorpio® (Stryker, Newbury, UK; *n*=46), PFC® Sigma® (DePuy, Leeds, UK; *n*=34) and NexGen® (Zimmer, Swindon, UK; *n*=22) (Table 1). All procedures were performed under general anaesthesia with the addition of femoral nerve blockade. The tourniquet was deflated after closure of the wound, application of dressings and a compression bandage with the knee in extension. Routine deep vein thrombosis prophylaxis comprised thromboembolic deterrent stockings, intra-operative calf pumps, early mobilisation and aspirin 150mg daily for six weeks unless contraindicated.

**Table 1:** Types of prosthesis in each group

	No drain	Retransfusion	Closed suction	Total
Scorpio®	29	6	11	46
PFC®	10	12	12	34
NexGen®	0	15	7	22
**Total**	**39**	**33**	**30**	**102**

In the first half of the study period the senior author used drains in all TKRs. A closed suction drain was chosen for patients over 70kg with pre-operative haemoglobin (Hb) levels over 13g/dl. An unwashed cell salvage drain system was used for patients under 70kg or if pre-operative Hb was under 13g/dl. During the second half of the study period the senior author used no drains regardless of patient weight or Hb level.

Where used, two drains were sited in the suprapatellar pouch immediately prior to wound closure. All drains were removed at 24 hours after surgery. Drainage in excess of 100ml into cell salvage drains during the first four hours was retransfused. Any blood collected more than four hours after surgery was discarded. Post-operatively, all patients displaying clinical signs of anaemia or with Hb levels below 8.0g/dl were transfused with allogenic packed red cells.

Knee outcome data in the form of the Oxford knee score (OKS) and American Knee Society score (AKSS) for knee and function scores were collected prospectively both pre-operatively and at subsequent follow-up. Case notes were studied retrospectively for data on sex, pre and post-operative Hb levels, number of units of allogenic red cells transfused and amount of retransfused blood.

### Criteria for scoring AKSS and OKS

The AKSS and OKS are both used widely and have been validated extensively.^7,8^ The AKSS comprises a knee score (out of 100) and a function score (out of 100). The AKSS form is completed by a member of the operative team while the OKS questionnaire is completed by the patient independently. In this study the OKS was used with 0 as the best possible and 48 as the worst possible scores.

### Statistical analysis

SPSS® 14.0 (SPSS, Chicago, IL, US) was used to analyse the data. Data were tested for normality by drawing histograms as well as with the Shapiro–Wilk test. The threshold for statistical significance was *p*<0.05 were considered to be significant. A one-way analysis of variance (ANOVA) was used to compare the means. Tukey and Scheffé post-hoc tests were performed in all cases to check for difference between the groups.

## Results

The ‘no drain’ group consisted of 39 patients, the ‘closed suction’ group of 30 patients and the ‘retransfusion’ group of 33 patients. There were 27 men and 75 women. There were no cases of infection in the series. One patient in the no drain group had a post-operative fracture. In view of this, she was included when calculating pre and post-operative Hb levels but excluded when calculating knee outcome scores as those were not recorded due to the complication. One patient in the retransfusion group was lost to follow-up and was therefore excluded from the knee outcome score calculations.

### Haemoglobin levels and transfusion rates

**Figure 1:**
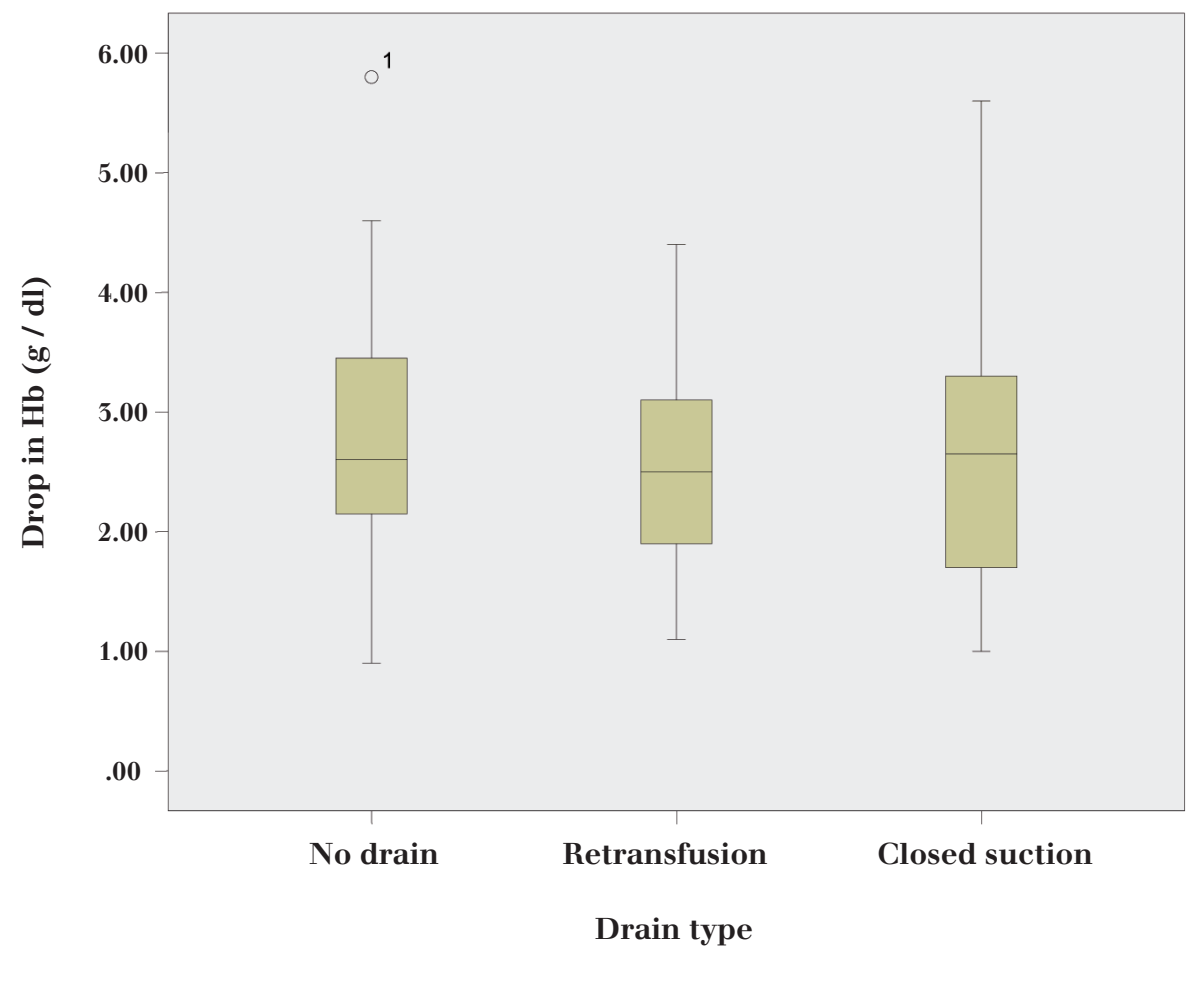
Box plot illustrating no difference in the drop in haemoglobin (Hb) levels between the groups

The total mean pre-operative Hb was 13.50g/dl and the mean post-operative Hb was 10.80g/dl (Table 2). The Shapiro–Wilk test showed a normal distribution of the mean drop in Hb levels (no drain: *p*=0.134, retransfusion: *p*=0.479, closed suction: *p*=0.368). There was no statistically significant difference in the drop in Hb levels between the groups (one-way ANOVA, *p*=0.41) (Fig 1).

**Table 2:** Results of haemoglobin (Hb) levels and transfusion rates of groups

	No drain	Closed suction	Retransfusion	Total
Men	11	6	10	27
Women	28	24	23	75
Male-to-female ratio	1:2.5	1:4	1:2.3	1:2.8
Mean pre-operative Hb (g/dl)	13.57 95% CI: 13.11–14.04	13.35 95% CI: 12.89–13.81	13.55 95% CI: 13.03–14.07	13.50 95% CI: 13.23–13.77
Mean post-operative Hb (g/dl)	10.73 95% CI: 10.28–11.18	10.63 95% CI: 10.04–11.22	11.02 95% CI: 10.47–11.52	10.80 95% CI: 10.50–11.09
Mean Hb drop (g/dl)	2.84 95% CI: 2.53–3.15 Range: 0.9–5.80	2.72 95% CI: 2.31–3.14 Range: 1.00–5.60	2.53 95% CI: 2.21–2.85 Range: 1.10–4.40	2.71 95% CI: 2.51–2.90 Range: 0.90–5.80
Units transfused post-operatively	6 units between 3 patients (2,2,2)	8 units between 3 patients (2,2,4)	6 units between 2 patients (3,3)	20 units between 8 patients
Retransfusion amount (ml)	–	–	504	504

CI = confidence interval

Overall, 20 units were transfused to 8 patients. In the no drain group, 3 (8%) of the 39 patients were transfused a total of 6 units. In the closed suction group, 3 (10%) of 30 patients were transfused a total of 8 units. In the retransfusion group, 2 (6%) of 33 patients were transfused a total of 6 units. Chi-squared analysis revealed no statistical difference in transfusion rates between the groups.

There were 33 patients in the retransfusion group. In this group, 30 patients (91%) successfully received autologous blood. A total of 16,140ml of blood was retransfused. The mean retransfusion volume was 504ml.

### OKS and AKSS

**Figure 2:**
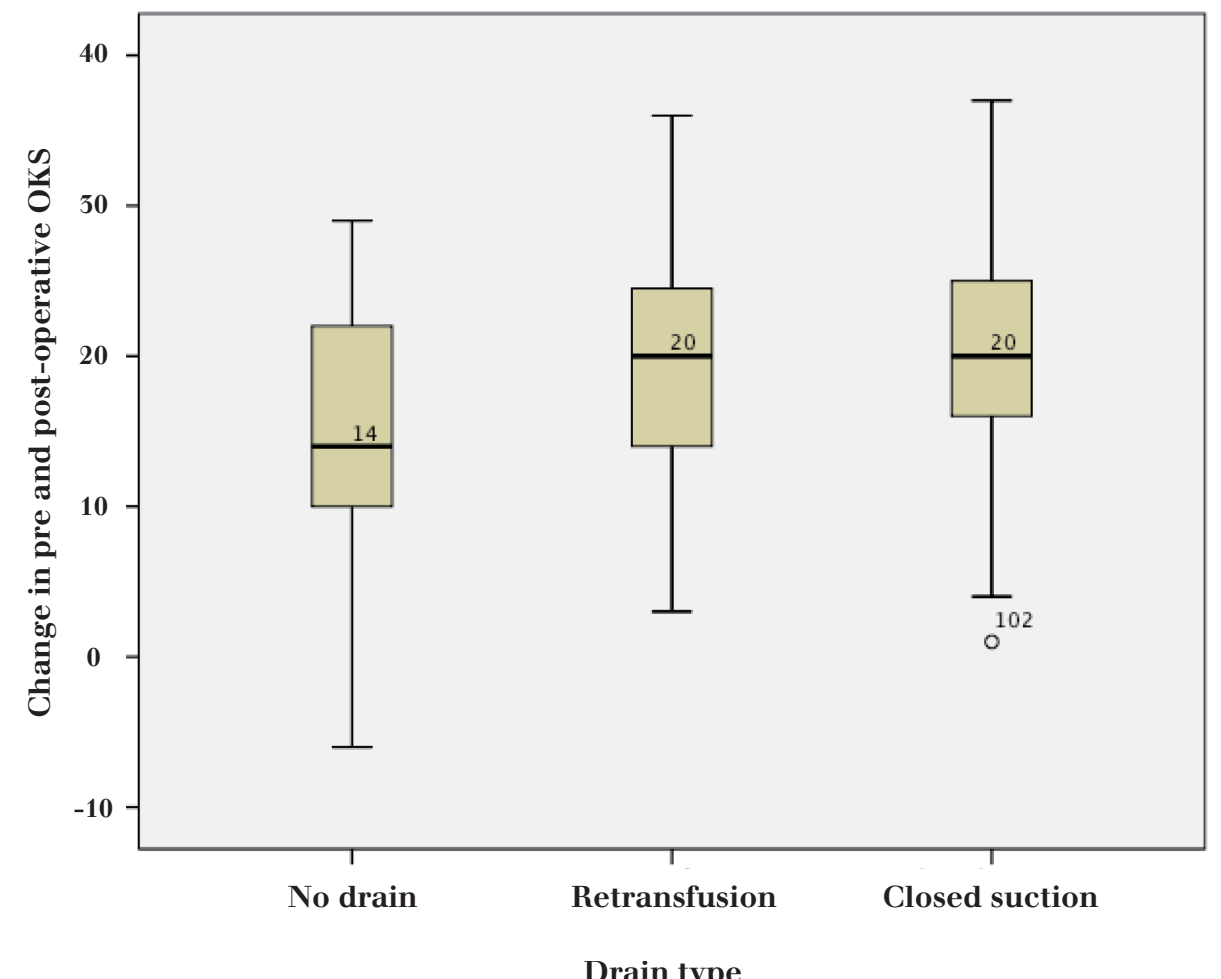
Box plot illustrating a reduced improvement in Oxford knee scores (OKS) in the ‘no drain’ group compared with the other groups

The Shapiro–Wilk test revealed normally distributed data for the difference between pre-operative and post-operative functions with each of the three scoring systems (OKS *p*=0.65, AKSS knee *p*=0.436, AKSS function *p*=0.119). There was a reduced improvement in the OKS in the no drain group compared with the other two groups following TKR (one-way ANOVA, *p*=0.013) (Fig 2). There was no statistically significant change in the AKSS knee and function scores between the groups following TKR.

## Discussion

Controversy still surrounds the use of drains after TKR. Most previous studies have only compared two groups (for example, suction drains vs reinfusion drains or no drains vs suction drains). We are aware of only one previous study that has compared all three groups. Adalberth *et al* randomised 90 patients into three groups comparing closed suction drains, retransfusion drains and no drains.^6^ There was no statistically significant difference in blood loss, range of knee motion or knee swelling between the three groups. Knee outcome scores and transfusion rates were not recorded, however.

Four previous studies were identified comparing retransfusion and closed suction drains.^5,9–11^ Kirkos *et al* found retransfusion drains gave higher post-operative Hb levels and lower rates of blood transfusion.^5^ However, Abuzakuk *et al* found no clinically significant difference between the groups.^9^ Jones *et al* showed that retransfusion drains required lower rates of autologous transfusion but with no difference in post-operative Hb levels.^10^ Lakshmanan *et al* found no difference in autologous transfusion rates or Hb levels post-operatively between the two groups.^11^

When comparing closed suction drains with no drains, three previous studies were identified.^12–14^ Mengal *et al* found no difference in post-operative Hb levels but higher rates of blood transfusion were required in the no drain group.^12^ Kumar *et al*^13^ and Sundaram and Parkinson^14^ both failed to show a statistically significant difference between the two groups using post-operative Hb levels and autologous transfusion rates as outcomes.

One study was identified comparing retransfusion drains with no drains. Jones *et al* found no statistically significant difference between the two groups in post-operative Hb levels or autologous blood transfusion rates.^15^

The results of our study confirm those of Adalberth *et al*^6^ in finding no statistically significant difference in post-operative Hb levels between the three groups and the requirement for allogenic blood transfusion was comparable in all groups. Our study goes further, however, to include the clinical outcome of these patients as measured by the OKS and AKSS. We are not aware of any previous study that has addressed this issue. Functionally, there was a small but statistically significant difference in OKS with the no drain group improving slightly less post-operatively. The clinical significance of this difference is doubtful, particularly when considering that there was no difference in the improvement in both the AKSS knee and function scores between the groups.

The strength of our study lies in the minimisation of confounding variables. The patients in this single surgeon series were recruited over a short time and post-operative care was identical in all cases. The weaknesses of our study lie in the retrospective analysis and non-randomised study design. Due to the relatively small number of patients involved there is a possibility of a type II error masking real differences between the groups. To address this limitation of our study and other similar studies, a larger prospective randomised study would be required. With the numbers available, we were unable to demonstrate a difference in transfusion requirements or clinical outcomes between the three study groups.

## Conclusions

Our study, which examined Hb levels, blood transfusion requirements and functional scores, does not support the use of either closed suction drains or reinfusion drains after primary elective TKR. Larger randomised trials are needed to confirm the findings of our study.

## References

[1] Kalairajah Y, Simpson D, Cossey AJ*et al.* Blood loss after total knee replacement: effects of computer-assisted surgery. J Bone Joint Surg Br 2005; 87: 1,480–1,482 16260662 10.1302/0301-620X.87B11.16474

[2] Padala PR, Rouholamin E, Mehta RL. The role of drains and tourniquets in primary total knee replacement: a comparative study of TKR performed with drains and tourniquet versus no drains and adrenaline and saline infiltration. J Knee Surg 2004; 17: 24–27 14971670 10.1055/s-0030-1247143

[3] Ong SM, Taylor GJ. Can knee position save blood following total knee replacement? Knee 2003; 10: 81–85 12649032 10.1016/s0968-0160(02)00076-5

[4] Tsumara N, Yoshiya S, Chin T*et al.* A prospective comparison of clamping the drain or post-operative salvage of blood in reducing blood loss after total knee arthroplasty. J Bone Joint Surg Br 2006; 88: 49–53 16365120 10.1302/0301-620X.88B1.16653

[5] Kirkos JM, Krystallis CT, Konstantinidis PA*et al.* Postoperative re-perfusion of drained blood in patients undergoing total knee arthroplasty: is it effective and cost-efficient? Acta Orthop Belg 2006; 72: 18–23 16570889

[6] Adalberth G, Byström S, Kolstad K*et al.* Postoperative drainage of knee arthroplasty is not necessary: a randomized study of 90 patients. Acta Orthop Scand 1998; 69: 475–478 9855227 10.3109/17453679808997781

[7] Insall JN, Dorr LD, Scott RD, Scott WN. Rationale of the Knee Society clinical rating system. Clin Orthop Relat Res 1989; 248: 13–14 2805470

[8] Dawson J, Fitzpatrick R, Murray D, Carr A. Questionnaire on the perceptions of patients about total knee replacement. J Bone Joint Surg Br 1998; 80: 63–69 9460955 10.1302/0301-620x.80b1.7859

[9] Abuzakuk T, Senthil Kumar V, Shenava Y*et al.* Autotransfusion drains in total knee replacement. Are they alternatives to homologous transfusion? Int Orthop 2007; 31: 235–239 16761149 10.1007/s00264-006-0159-y PMC2267563

[10] Jones HW, Savage L, White C*et al.* Postoperative autologous blood salvage drains – are they useful in primary uncemented hip and knee arthroplasty? A prospective study of 186 cases. Acta Orthop Belg 2004; 70: 466–473 15587036

[11] Lakshmanan P, Purushothaman B, Sharma A. How beneficial are re-infusion drains in total knee arthroplasty. Transfus Med 2008; 18: 74–75 18279195 10.1111/j.1365-3148.2007.00814.x

[12] Mengal B, Aebi J, Rodriquez A, Lemaire R. A prospective randomized study of wound drainage versus non-drainage in primary total hip or knee arthroplasty. Rev Chir Orthop Reparatrice Appar Mot 2001; 87: 29–39 11240535

[13] Kumar S, Penematsa S, Parekh S. Are drains required following a routine primary total joint arthroplasty? Int Orthop 2007; 31: 593–596 17033763 10.1007/s00264-006-0245-1 PMC2266645

[14] Sundaram RO, Parkinson RW. Closed suction drains do not increase the blood transfusion rates in patients undergoing total knee arthroplasty. Int Orthop 2007; 31: 613–616 16947048 10.1007/s00264-006-0232-6 PMC2266643

[15] Jones AP, Harrison M, Hui A. Comparison of autologous transfusion drains versus no drain in total knee arthroplasty. Acta Orthop Belg 2007; 73: 377–385 17715730

